# Supplementary material for: Identifying and describing alcohol-related paediatric emergency department attendances amongst under 16 year olds including time trends, incidence rates, and sociodemographic factors associated with alcohol-related harm
Source: PLoS One. 2025 Aug 19;20(8):e0329502. doi: 10.1371/journal.pone.0329502 (PMC12364363; doi:10.1371/journal.pone.0329502)
Supplement: S1 Table — (DOCX) [file pone.0329502.s001.docx]

S1 Table. Number of alcohol-related attendances per year and percentage admitted to hospital, by gender.

| **Year** | **n male attendances** | **n female attendances** | **% admitted male** | **% admitted female** |
| --- | --- | --- | --- | --- |
| 2011 | 43 | 77 | 37.21 | 32.47 |
| 2012 | 28 | 59 | 28.00 | 20.34 |
| 2013 | 22 | 46 | 50.00 | 32.61 |
| 2014 | 11 | 53 | 54.55 | 32.08 |
| 2015 | 16 | 32 | 31.25 | 21.88 |
| 2016 | 18 | 43 | 33.33 | 23.26 |
| 2017 | 20 | 66 | 25.00 | 22.73 |
| 2018 | 11 | 50 | 45.45 | 28.00 |
| 2019 | 15 | 63 | 26.67 | 15.87 |
| 2020 | 5 | 30 | 20.00 | 20.00 |
| 2021 | 11 | 47 | 27.27 | 21.28 |
| 2022 | 14 | 32 | 7.14 | 12.50 |
