# Supplementary material for: Identifying and describing alcohol-related paediatric emergency department attendances amongst under 16 year olds including time trends, incidence rates, and sociodemographic factors associated with alcohol-related harm
Source: PLoS One. 2025 Aug 19;20(8):e0329502. doi: 10.1371/journal.pone.0329502 (PMC12364363; doi:10.1371/journal.pone.0329502)
Supplement: S2 Table — (DOCX) [file pone.0329502.s002.docx]

**S2 Table. Proportion of alcohol-related attendances per year compared to mid-year population estimates for 5 to 15 year olds in Merseyside (by gender).**

| Year | Alcohol-related Attendances (n) | | ONS Population estimate aged 5 to 15 years old (n) | | Attendance rates per 100,000 [95%CI] | |
| --- | --- | --- | --- | --- | --- | --- |
|  | Male | Female | Male | Female | Male | Female |
| 2011 | 43 | 77 | 84888 | 81197 | 50.65 [37.61, 68.22] | 94.83 [73.66, 116.00] |
| 2012 | 28 | 59 | 84280 | 80603 | 33.22 [22.99, 48.01] | 73.20 [56.76, 94.40] |
| 2013 | 22 | 46 | 83768 | 80123 | 26.26 [17.35, 39.76] | 57.41 [40.83, 74.00] |
| 2014 | 11 | 53 | 83784 | 80540 | 13.13 [7.33, 23.51] | 65.81 [50.32, 86.06] |
| 2015 | 16 | 32 | 84285 | 80808 | 18.98 [11.69, 30.84] | 39.60 [28.05, 55.90] |
| 2016 | 18 | 43 | 85441 | 81478 | 21.07 [13.33, 33.50] | 52.77 [39.19, 71.07] |
| 2017 | 20 | 66 | 86933 | 82799 | 23.01 [14.89. 35.53] | 79.71 [62.67, 101.39] |
| 2018 | 11 | 50 | 88233 | 83902 | 12.47 [6.96, 22.32] | 59.59 [45.21, 78.55] |
| 2019 | 15 | 63 | 89120 | 84655 | 16.83 [10.20, 27.77] | 74.42 [58.18, 95.19] |
| 2020 | 5 | 30 | 89657 | 85056 | 5.58 [2.38, 13.06] | 35.27 [24.71, 50.35] |
| 2021 | 11 | 47 | 90219 | 85271 | 12.19 [6.81, 21.83] | 55.12 [41.46, 73.28] |
| 2022 | 14 | 32 | 91774 | 86710 | 15.25 [9.09, 25.61] | 36.90 [26.14, 52.09] |
